# Deficiency of Activating Fcγ-Receptors Reduces Hepatic Clearance and Deposition of IC and Increases CIC Levels in Mercury-Induced Autoimmunity

**DOI:** 10.1371/journal.pone.0013413

**Published:** 2010-10-15

**Authors:** Klara Martinsson, Thomas Skogh, Seyed Ali Mousavi, Trond Berg, Jan-Ingvar Jönsson, Per Hultman

**Affiliations:** 1 Division of Molecular and Immunological Pathology (AIR), Department of Clinical and Experimental Medicine, Linköping University, Linköping, Sweden; 2 Rheumatology Unit (AIR), Department of Clinical and Experimental Medicine, Linköping University, Linköping, Sweden; 3 Experimental Hematology Unit, Department of Clinical and Experimental Medicine, Linköping University, Linköping, Sweden; 4 Medical Genetics Laboratory, Department of Medical Genetics, Rikshospitalet University Hospital, Oslo, Norway; 5 Department of Molecular Biosciences, University of Oslo, Oslo, Norway; Institut Jacques Monod, France

## Abstract

**Background:**

Inorganic mercury (Hg) induces a T-cell dependent, systemic autoimmune condition (HgIA) where activating Fcγ-receptors (FcγRs) are important for the induction. In this study we examined the influence of activating FcγRs on circulating levels and organ localization of immune complexes (IC) in HgIA.

**Methods and Principal Findings:**

Mercury treated BALB/c wt mice showed a significant but modest increase of circulating IC (CIC) from day 12 until day 18 and day 35 for IgG2a- and IgG1- CIC, respectively. Mercury-treated mice lacking the trans-membrane γ-chain of activating FcγRs (FcRγ^−/−^) had significantly higher CIC levels of both IgG1-CIC and IgG2a-CIC than wt mice during the treatment course. The hepatic uptake of preformed CIC was significantly more efficient in wt mice compared to FcγR^−/−^ mice, but also development of extrahepatic tissue IC deposits was delayed in FcRγ^−/−^ mice. After 35 days of Hg treatment the proportion of immune deposits, as well as the amounts was significantly reduced in vessel FcRγ^−/−^ mice compared to wt mice.

**Conclusions:**

We conclude that mice lacking functional activating FcγRs respond to Hg with increased levels and altered quality of CIC compared with wt mice. Lack of functional activating FcγRs delayed the elimination of CIC, but also significantly reduced extrahepatic tissue localization of CIC.

## Introduction

The deposits of glomerular immune complexes (IC) is a hallmark of certain systemic autoimmune diseases with glomerulonephritis (GN) [1]. However, the formation of ICs is also a physiological function of the immune system in order to eliminate antigens and to regulate immune responses [2], [3] . IgG-containing circulating ICs (CIC) are cleared via Fc-gamma receptor (FcγR) dependent uptake by Kupffer cells as well as liver sinusoidal endothelial cells [4]–[8]. In addition, hepatic elimination and extrahepatic deposition of CIC are affected by complement and complement receptors [9], [10]. If the physiological mechanisms of hepatic IC-elimination fails, extrahepatic tissue deposition of IC may occur and lead to tissue inflammation and organ damage [1]. The damage following tissue IC deposits depends on the mechanism and site of formation, but especially on the amount of deposits and their composition [1]. Thus, tissue ICs in systemic inflammatory disease may be derived from the circulation, as indicated by murine autoimmune models [11], [12], and in human diffuse proliferative lupus nephritis [13], or membranous GN [14]. The amount of CIC correlates with disease severity in systemic lupus erythematosus (SLE), where patients with overt nephritis show higher levels of CIC than patients with silent nephritis [15], [16]. Tissue IC deposits may, however, also form *in situ*, either by the interaction of antibodies (abs) with antigens planted in the glomerulus, or by binding of abs to intrinsic glomerular antigens [17], [18]. Formation of tissue IC deposits in SLE may be due to abnormal handling by FcγRs since hepatic FcγR-mediated IC clearance is less efficient in SLE patients than in healthy individuals [19].

All murine FcγRs ligate exposed Fcγ parts of ICs via the surface-exposed γ-chain: FcγRI, FcγRIII and FcγRVI, but not the inhibitory FcγRIIb, then promote cell activation via a signal-transducing trans-membrane dimeric γ-chain (FcRγ) residing the immune receptor tyrosine-based activation motif (ITAM) [20]. Furthermore, the trans-membrane FcRγ is essential for endocytosis of surface-bound soluble IgG-ICs and phagocytosis of IgG-opsonized particles via the stimulating FcγRs, although Fc-ligation remains intact [20], [21]. FcγRIII is the predominant receptor in triggering immune effector functions following murine IgG1-IC binding [22]. The response is also influenced by the inhibitory FcγRIIb, where the immune receptor tyrosine-based inhibitory motif (ITIM) becomes phosphorylated upon Fc-ligation, thereby inhibiting ITAM- signalling [20]. In rats, the FcγRIIb2 of liver sinusoidal endothelial cells (LSEC) is used not only as a receptor for efficient CIC clearance, but also as a recycling receptor with or without ligated ICs [8].

Lack of FcγRIIB increases the incidence of nephritis in murine pristane-induced lupus [23], and may even cause spontaneous GN in some mouse strains [24]. A delicate balance between activating and inhibitory signals emanating from the FcγRs characterizes a normal immune response. This balance may be disturbed in autoimmune diseases [2], [3], [25].

The importance of the activating FcγRs (FcγRI, FcγRIII and FcγRIV in mice) and the inhibiting FcγRIIb in autoimmune diseases has been elucidated in mouse strains with targeted knockout mutations for these FcγRs [25], [26]. We have previously used FcγR-deficient mice to explore the role of these receptors in HgIA [27], [28], a model characterized by Hg-induced lymphoproliferation, hypergammaglobulinaemia, antinucleolar autoantibodies (ANoA) and IC deposits in the renal glomerular mesangium and systemically in vessel walls in susceptible mouse strains [29]. In HgIA, activating FcγRs affect development of ANoA [28] as well as IC deposits [27], while the inhibiting FcγRIIb down-regulates the hyper-gammaglobulinaemic response [27], [28].

In this study we aimed at elucidating the effect of the signal-transducing trans-membrane dimeric γ-chain of activating FcγRs in HgIA on levels of CIC, hepatic IC uptake, and the development and composition of IC deposits in typical target organs in systemic autoimmune disease.

## Results

Firstly, we compare the levels of CIC in wildtype (wt) and FcRγ^−/−^ BALB/c mice in relation to the development of tissue IC deposits during five weeks of Hg treatment. Secondly, we report differences regarding blood clearance and hepatic uptake of preformed model immune complexes in the two BALB/c strains. Thirdly, we analyse the composition of tissue IC deposits in the two mouse strains after five weeks of Hg treatment.

### Increased levels of IgG1- and IgG2a-containing CIC are associated with development of high-titred tissue IC deposits in wt BALB/c mice but not in FcRγ^−/−^ mice

#### Wt mice

**Figure 1:**
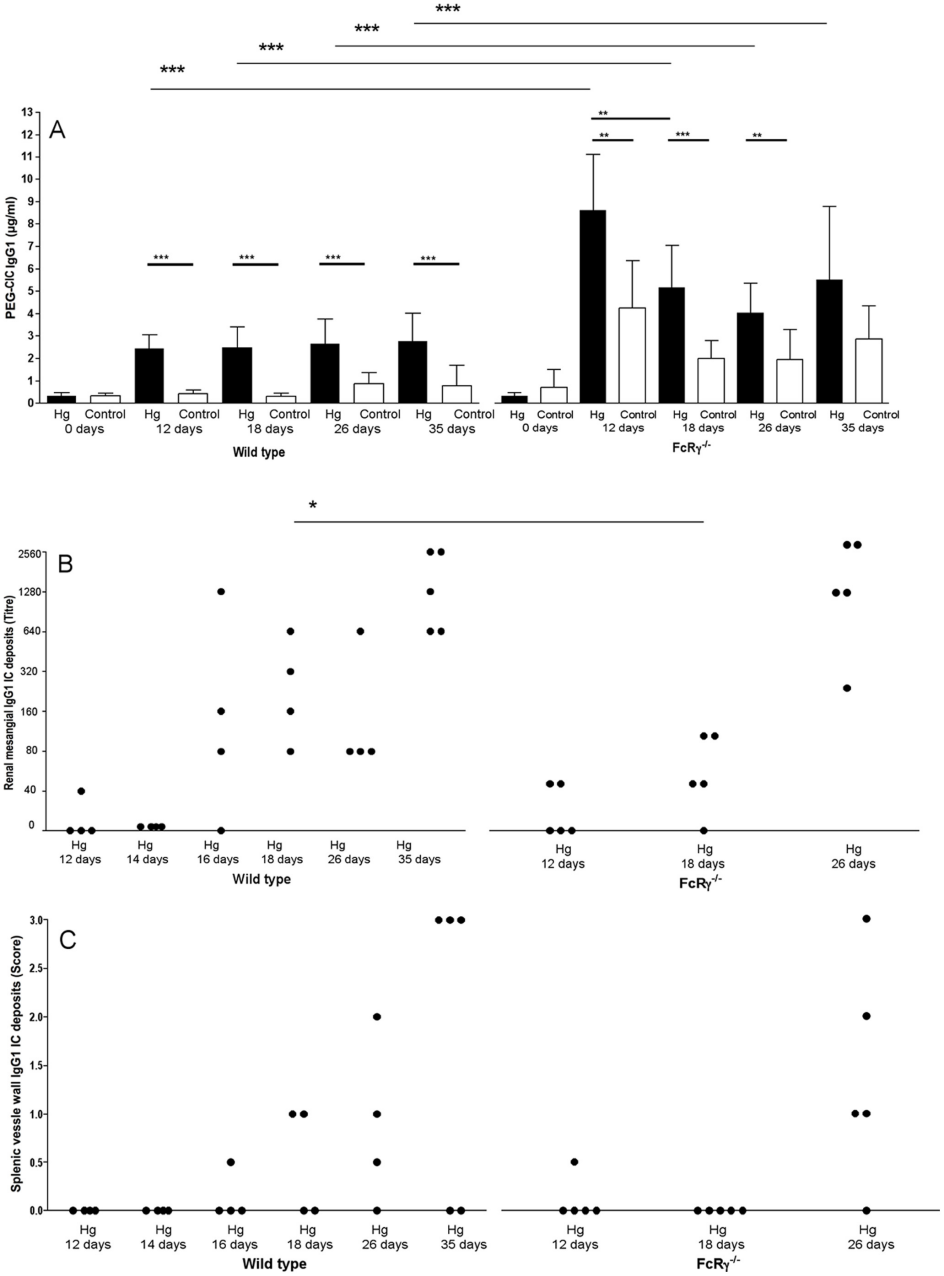
Development of circulating IgG1 immune complexes and tissue IgG1 immune complex deposits. Development of circulating IgG1-containing immune complexes and tissue IgG1 immune complex deposits during 26–35 days of treatment of female BALB/c wild type and FcRγ^−/−^ mice with 15 mg/L HgCl_2_ in the drinking water or drinking water without any addition of Hg (controls). (A) PEG-precipitated circulating immune complexes containing IgG1 antibodies. The bars denote mean ± SD. ** p<0.01 and *** *p<0.001* (Mann-Whitney's test). (B) Renal mesangial IgG1 and (C) splenic vessel wall IgG1 deposits. Each symbol represents a single mouse. * p<0.05 (Mann-Whitney's test).

**Figure 2:**
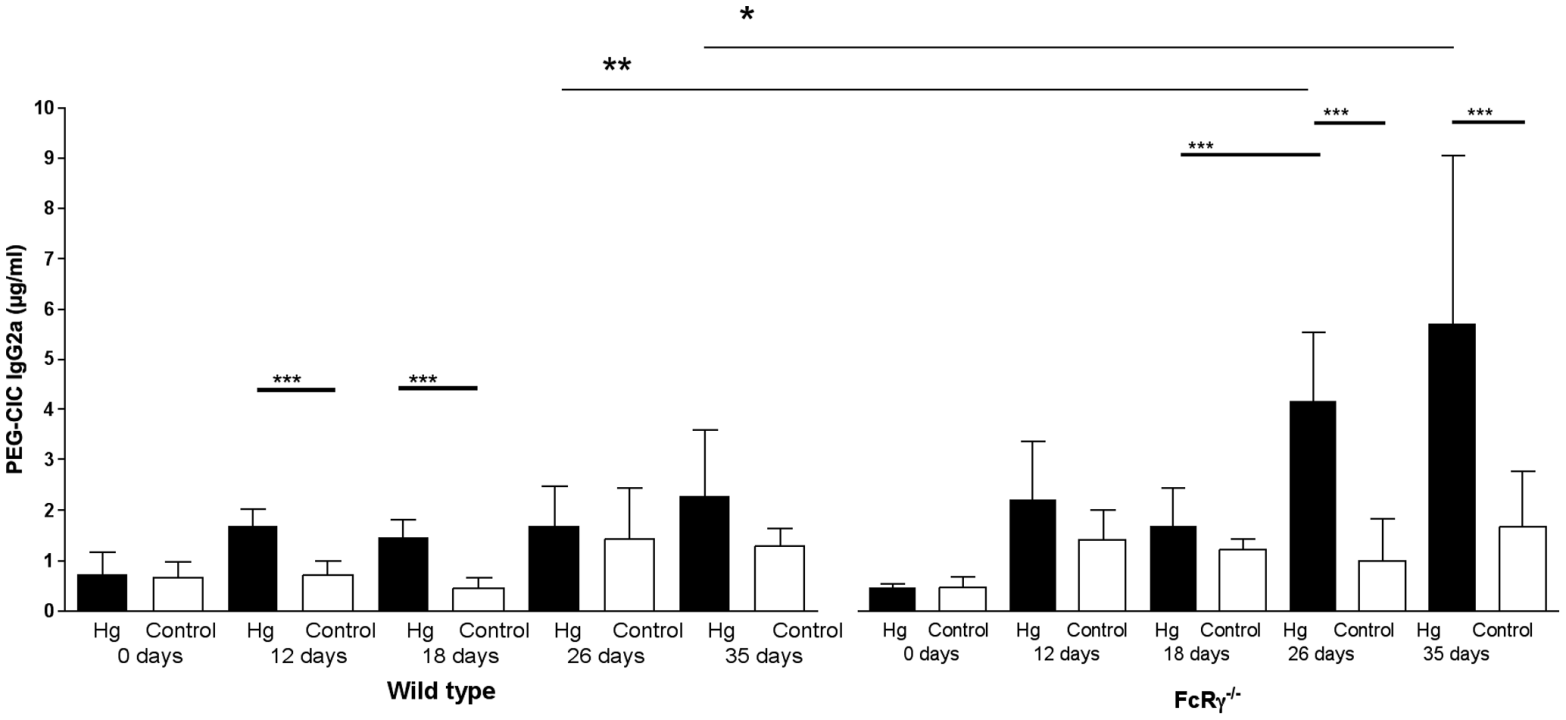
Development of circulating IgG2a immune complexes. Development of PEG-precipitated circulating IgG2a-containing immune complexes during 26–35 days of treatment of female BALB/c wild type and FcRγ^−/−^ mice with 15 mg/L HgCl_2_ in the drinking water or drinking water without any addition of Hg (controls). The bars denote mean ± SD. * p<0.05, ** p<0.01 and *** *p<0.001* (Mann-Whitney's test).

BALB/c wt mice treated with Hg had significantly higher concentrations of CIC containing IgG1 (Figure 1A) and IgG2a (Figure 2) after 12 (*p<0.001*) and 18 (*p<0.001*) days of treatment, and regarding IgG1-CIC after 26 (*p<0.001*) and 35 (*p<0.001*) days as compared to untreated mice. Renal mesangial (Figure 1B) and splenic vessel wall (Figure 1C) IgG1 deposits were first seen after 16 days of Hg treatment, and the fraction of mice with IC deposits and/or the titre of the deposits increased until end of treatment after 35 days. Despite the increase of IgG2a-CIC in Hg-treated wt mice (Figure 2) neither renal mesangial nor splenic vessel wall deposits contained IgG2a (data not shown). While C3c deposits were seen already after 12 days treatment in the renal mesangium, C3c deposits were not seen in splenic vessel walls until the end of treatment after 35 days of Hg treatment (data not shown). None of the untreated wt mice showed IgG1, IgG2a or C3c deposits in glomeruli or vessel walls at any time during the 35 days of Hg treatment (data not shown).

#### FcRγ^−/−^ mice

In FcRγ^−/−^ mice the levels of IgG1-containing CIC were highest after 12 days of Hg treatment (Figure 1A), but remained significantly increased compared to untreated FcRγ^−/−^ mice during an additional 14 days of treatment (for 12 days *p<0.01*, 18 days *p<0.001* and 26 days *p<0.01*). The IgG1-CIC level was significantly higher in Hg-treated FcRγ^−/−^ mice as compared to wt mice during all of the 35 days (12 days *p<0.001*, 18 days *p<0.001*, 26 days *p<0.01* and 35 days *p<0.05*). In the FcRγ^−/−^ mice, traces of renal mesangial (Figure 1B) and splenic vessel wall (Figure 1C) IgG1 deposits were seen after 12–18 days of Hg treatment, but higher titres of IgG1 deposits were not seen until 26 days of treatment (Figure 1B–C). After 18 days of Hg treatment the titre of IgG1 deposits in the renal mesangium of FcRγ^−/−^ mice was significantly lower than in wt mice (*p<0.05*).

The level of IgG2a-containing CIC was significantly increased in FcRγ^−/−^ mice after 26 and 35 days of Hg treatment compared to both untreated FcRγ^−/−^ mice (*p<0.001*) and Hg-treated wt mice (26 days *p<0.01* and 35 days *p<0.05*, respectively) (Figure 2). However, IgG2a deposits were not detected in renal mesangium or splenic vessel walls at any time (data not shown). C3c deposits first appeared in the renal mesangium of Hg-treated FcRγ^−/−^ mice after 12 days but with significantly lower titres than in wt mice (*p<0.05*) (data not shown). C3c deposits were seen in splenic vessel wall of Hg-treated FcRγ^−/−^ mice after 26 days, and the fraction of positive mice was significantly higher (*p<0.05*) than in Hg-treated wt mice. However, the amount of deposits was low with a score of 0.5±0.7 (mean ± SD). None of the untreated FcRγ^−/−^ mice developed IgG1, IgG2a or C3c deposits (data not shown).

The levels of C1q-binding CIC were measured in the above sera but there was no difference, neither between Hg-treated and untreated mice nor between wt and FcRγ^−/−^ mice (data not shown).

In conclusion, increased levels of IgG1- and IgG2a-containing CIC were associated with development of tissue IC deposits in Hg-treated wt BALB/c mice. While significantly higher levels of CIC were seen in FcRγ^−/−^ mice, the development of high-titred tissue IC deposits was delayed as compared to wt mice.

### Intact functional activating FcγRs are important for the elimination of CIC by the liver

**Figure 3:**
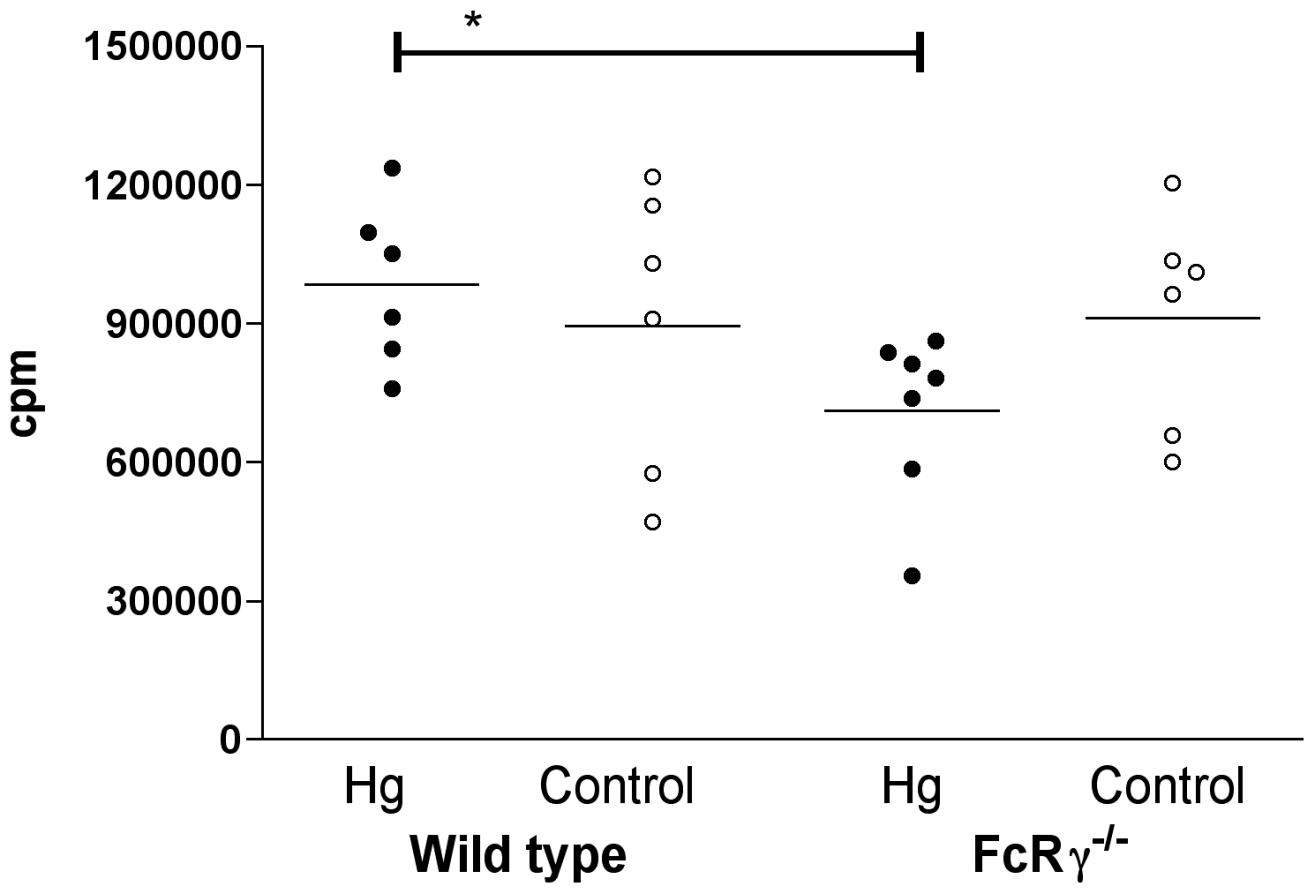
Uptake of circulating immune complexes in the liver. Uptake of preformed circulating HSA/DNP-IgG immune complexes in the liver of female BALB/c wild type and FcRγ^−/−^ mice following 15–17 days of treatment with 15 mg/L HgCl_2_ in the drinking water or drinking water without any addition of Hg (controls). The bars denote mean ± SD. * *p<0.05* (Mann-Whitney's test).

BALB/c wt and FcRγ^−/−^ mice treated with Hg for 15–17 days showed a similar clearance rate of preformed dinitrophenyl (DNP)- conjugated human serum albumin (HSA)/IgG IC. No difference was seen in clearance rate comparing Hg-treated and untreated mice (data not shown). In contrast, Hg-treated wt mice showed a significantly higher hepatic uptake of DNP-HSA/IgG IC compared to Hg-treated FcRγ^−/−^ mice (*p<0.05*) demonstrating the importance of intact functional activating FcγRs for the elimination of CIC by the liver (Figure 3).

### Lack of intact functional activating FcγRs reduces tissue IC deposits

#### Splenic vessel wall IC deposits in wt mice

**Figure 4:**
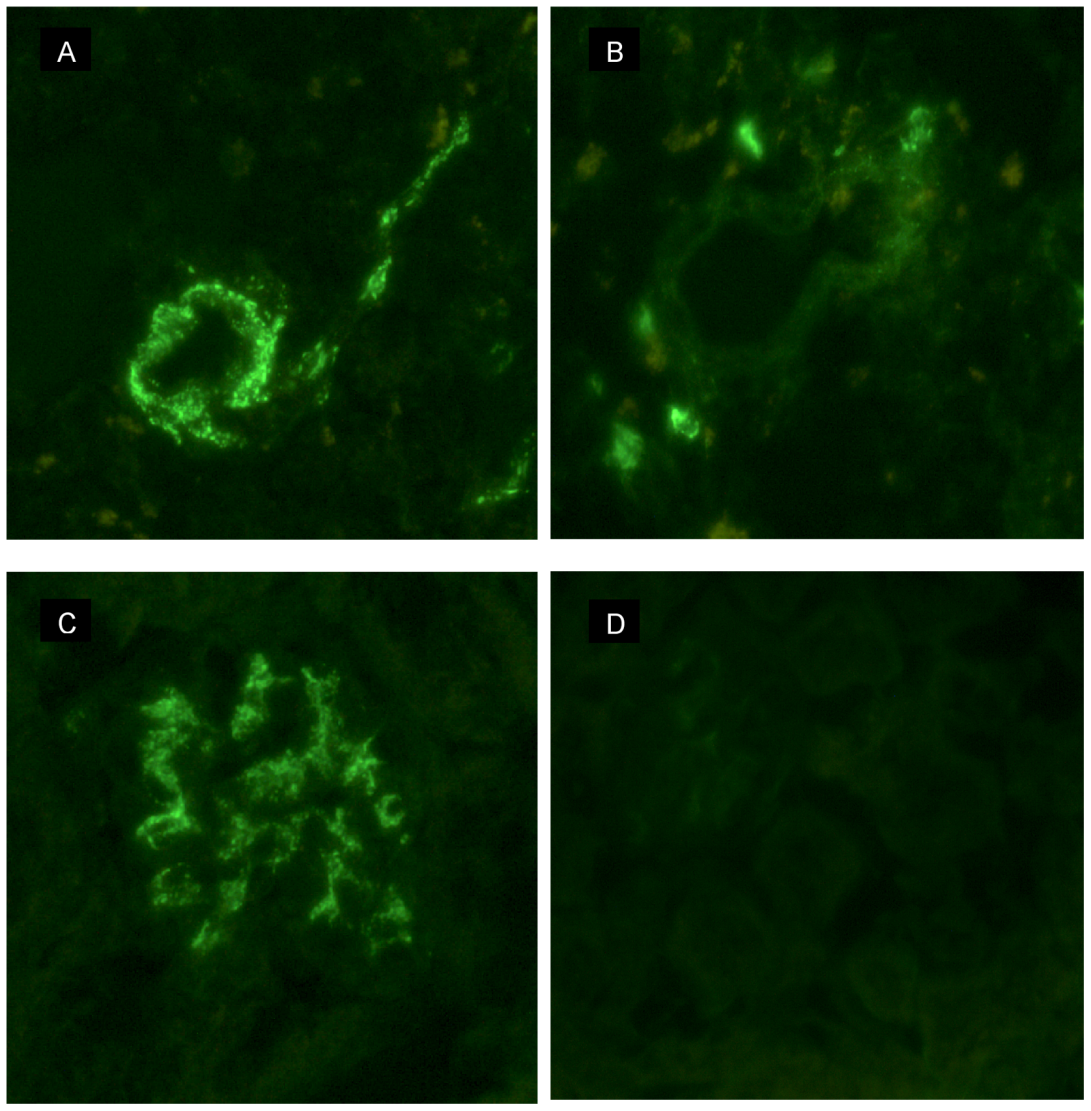
Tissue IgG1 immune complex deposits. Direct immunofluorescence using FITC-conjugated anti-IgG1 antibodies on cryostate sections from female BALB/c wild type (A, C) and FcRγ^−/−^ (B, D) mice treated with 15 mg/l HgCl_2_ in the drinking water for 5 weeks. Heavy granular staining in splenic vessel walls (A) and renal mesangium (C) in wild type mice, but only slight deposits in the corresponding tissues of FcRγ^−/−^ mice (B, D).

All nine wt mice showed IgG1-IC deposits in splenic vessel walls (Figure 4A) after 5 weeks of Hg treatment, while none of the untreated mice (*p<0.001*) showed such deposits (Table 1). Hg treatment caused slight deposits of IgG2b and IgG3 in one and two mice, respectively. The proportion of mice with C3c deposits in the splenic vessel walls was significantly higher (*p<0.001*) in Hg-treated mice than in untreated mice (Table 1). None of the wt mice showed C1q deposits (Table 1).

**Table 1:** Composition of immune complex deposits in splenic vessel walls from HgCl_2_-treated and untreated BALB/c mice after 5 weeks.

			Splenic vessel walls						
Strain	No	Treatment	IgG1	IgG2a	IgG2b	IgG3	Total IgG	C1q	C3c
Wt	9	Hg^a^	100b, c	0	11	22	100	0	100^c^
			(3.1±0.8)^d^		(0.1±0.3)	(0.2±0.4)			(2.1±0.6)
Wt	10	Untreated	0	0	ND	ND	0	0	0
FcRγ^−/−^	9	Hg^a^	89^e^	0	0	11	89	0	56f,g
			(1.7±1.1)^h^			(0.1±0.2)			(0.3±0.4)^i^
FcRγ^−/−^	10	Untreated	0	0	ND	ND	0	0	0

a 15 mg/L HgCl_2_ in the drinking water,

b Fraction of mice with immune complex deposits,

c significantly different from untreated wt mice (Fisher's exact test *p<0.001*),

d Grading, 0–4: figures denote mean ± SD,

e significantly different from untreated FcRγ^−/−^ mice (Fisher's exact test *p<0.001),*

f significantly different from untreated FcRγ^−/−^ mice (Fisher's exact test *p<0.05),*

g significantly different from Hg-treated wt mice (Fisher's exact test *p<0.05*),

h significantly different from Hg-treated wt mice (Mann-Whitney's test *p<*0.05),

i significantly different from Hg-treated wt mice (Mann-Whitney's test *p<0.001*)

Wt, wild type mice; ND, not determined.

#### Splenic vessel wall IC deposits in FcRγ^−/−^ mice

Eight out of 9 (89%) Hg-treated FcRγ^−/−^ mice had developed IgG1 deposits in the splenic vessel walls after 5 weeks (Figure 4B), which was significantly higher than in the untreated FcRγ^−/−^ mice (*p<0.001*) (Table 1). The amount of IgG1 deposits was significantly (*p<0.05*) lower in Hg-treated FcRγ^−/−^ compared to Hg-treated wt mice (Table 1). Fifty-six percent of the Hg-treated FcRγ^−/−^ mice showed C3c deposits in the splenic vessel walls, which was significantly higher than in untreated mice (*p<0.05)*. However, the percentage of mice with C3c deposits as well as the amount of deposits (*p<0.05*, *p<0.001*, respectively) were significantly lower in Hg-treated FcRγ^−/−^ mice than in wt mice (Table 1). None of the untreated FcRγ^−/−^ mice developed C3c deposits. Neither Hg-treated nor untreated FcRγ^−/−^ mice showed C1q deposits in the splenic vessel walls (Table 1).

#### Renal glomerular and vessel wall IC deposits in wt mice

After 5 weeks of Hg treatment renal mesangial IC deposits were dominated by IgG1 (Figure 4C), although most mice also showed a relatively low titre of IgG2b. IgG2a and IgG3 deposits were less frequent and of a low titre (Table 2). IgG1 and IgG2a deposits were not observed in untreated mice. The Hg-treated wt mice developed mesangial C3c deposits, which were present also in untreated mice but with only a low titre. None of the wt mice developed C1q deposits (Table 2).

**Table 2:** Composition of immune complex deposits in renal mesangial and vessel walls from HgCl_2_-treated and untreated BALB/c mice after 5 weeks.

			Renal mesangial IC deposits							Renal vessel wall IC deposits					
Strain	No	Treatment	IgG1	IgG2a	IgG2b	IgG3	Total IgG	C1q	C3c	IgG1	IgG2a	IgG2b	IgG3	C3c	C1q
Wt	9	Hg^a^	100^b^	22	89	67	100	0	100	11	0	0	0	0	0
			(1351±747)^c^	(13±28)	(169±123)	(44±37)			(391±198)	(0.1±0.3)^d^					
	10	Untreated	0	0	ND	ND	0	0	100	0	0	ND	ND	0	0
									(80±0)						
FcRγ^−/−^	9	Hg^a^	100^e^	25^e^	88^e^	13^e^	100	0	100^f^	25	0	0	0	0	0
			(620±470)^g^	(20±37)	(200±113)	(10±28)			(389±242)	(0.4±1)					
	10	Untreated	0	0	ND	ND	0	0	100	0	0	ND	ND	0	0
									(176±51)						

a 15 mg/L HgCl_2_ in the drinking water,

b Fraction of mice with immune complex deposits,

c Reciprocal titre, figures denote mean ± SD,

d Grading, 0–4, figures denote mean ± SD,

e results from 8 mice,

f results from 7 mice,

g significantly different from Hg-treated wt mice (Mann-Whitney's test *p<0.05*).

Wt, wild type mice; ND, not determined.

A single wt mouse treated with Hg but none of the untreated wt mice showed IC deposits of the IgG1 isotype in the renal vessel walls (Table 2). Neither Hg-treated nor untreated wt mice developed C1q or C3c renal vessel wall IC deposits.

#### Renal glomerular and vessel wall IC in FcRγ^−/−^ mice

The Hg-treated FcRγ^−/−^ mice also developed renal mesangial IC deposits mainly containing IgG1 and IgG2b (Table 2). The IgG1 titre was significantly lower in Hg-treated FcRγ^−/−^ mice than in Hg-treated wt mice (Figure 4D) (*p<0.05)* (Table 2). There was no significant difference in the titre of C3c deposits between Hg-treated and untreated FcRγ^−/−^ mice and none of the mice developed C1q deposits (Table 2). Two FcRγ^−/−^ mice treated with Hg developed IgG1 renal vessel wall deposits without C1q or C3c deposits (Table 2), whereas none of the untreated FcRγ^−/−^ mice showed IgG1, IgG2a, C1q or C3c deposits.

Taken together these results show that FcRγ^−/−^ mice develop less IgG1 and C3c deposits in the splenic vessel walls and lower IgG1 titre in the renal mesangium compared to Hg-treated wt mice.

## Discussion

The present study demonstrates that BALB/c mice with Hg-induced systemic autoimmunity respond with significantly increased concentrations of CIC containing IgG1 and IgG2a compared to untreated mice. Confirming previous results [12] we conclude that Hg treatment *per se* does not affect the elimination rate of CIC, suggesting that Hg-induced IC formation accounts for the raised levels of CIC. We also demonstrated that the concentration of IgG-CIC was significantly higher in FcRγ^−/−^ mice than in wt animals, and that this functional deficiency in trans-membrane signalling of activating FcγRs is associated with deficient hepatic clearance of circulating IgG-IC. This accords with the findings of Ahmed *et al*, who reported that reduced FcγR expression on hepatic non-parenchymal cells was associated with spontaneous development of IC-mediated-GN in NZB/WF1 mice [30]. In SLE reduced expression of FcγRIII and FcγRII is association with high levels of CIC, prolonged FcγR-mediated clearance and high disease activity [31]. Willocks *et al* reported that a low copy number of the human FCGR3B gene correlates with reduced neutrophil expression of FcγRIIIB as well as with reduced neutrophil adherence to and uptake of IC in SLE patients [32].

The levels of circulating IgG and IgG-containing IC depend on several factors *e.g.* (i) the rate of antibody production, which in turn depend on the balance between exposure of activating/inhibiting FcγRs [2], [3], (ii) elimination from the circulation via non-specific escape/tissue deposits [10], and (iii) FcγR-mediated binding and endocytosis or recirculation [3]–[5], [8]. It is likely that the increased CIC concentration in the Hg-treated FcRγ^−/−^ mice as compared to wt mice is caused by a disturbance of the normal hepatic IC clearance. The uptake of CIC was not completely lost in mice deficient for trans-membrane signalling by activating FcγRs. This may to some extent be explained by IC-adherence to the stimulating FcγRs although endocytosis was deficient, and to some extend by binding to and endocytosis via FcγRIIB2 exposed on liver sinuoisdal endothelial cells as shown in rats [8].

The profile of CIC increase in Hg-treated mice was quite different between wt and FcRγ^−/−^ mice, the former showing a modest but steady increase from day 12, while the latter showed an initially 4-fold higher concentrations of IgG1-CIC on day 12 which subsequently declined but with a vigorous increase of IgG2a-CIC on day 26–35. FcRγ^−/−^ mice did not respond with a rise of serum IgG1 following Hg treatment as seen in wt mice [27], [28] indicating another mechanism for the elevation of CIC levels. The higher and also more variable CIC levels in the Hg-treated FcRγ^−/−^ mice might be due both to the reduced ability by these mice to handle normal levels of CIC and to the extensive Hg-induced IC-production. The higher concentration of CIC of first IgG1 and then IgG2a in Hg-treated FcRγ^−/−^ mice was associated with a delayed and reduced deposition of IgG1 IC in the renal mesangium as well as IgG1 and C3c systemically in vessel walls.

IC deposits can be generated from preformed CIC or by *in situ* formation due to adsorption of circulating abs to antigen exposed in the tissue [11], [12], [18]. The immune deposits seen in Hg-treated wt BALB/c mice may thus originated from serum IgG binding either to antigen planted in the tissue or to endogenouse tissue antigen, or from tissue deposition/binding of IgG-containing CIC. There are observations indicating that formation of IC, containing nucleosomes as antigens, take place within the kidney or in the circulation prior to deposition [33]–[35]. The only identified autoantigen in HgIA is fibrillarin (AFA) [36], which has also been indicated to be present in murine IC deposits [37]. However, the presence of AFA is not necessarily followed by IC formation [38], [39]. On the other hand, IC deposits may develop without formation of AFA as seen in Hg-treated BALB/c mice [27], [40]. Anti-nuclear abs (ANA), especially anti-nucelosomal have been implicated to be important in lupus nephritis [33]–[35]. Although anti-nucleosomal abs are seldom induced by Hg treatment of BALB/c mice, we have previously reported that ANA of IgG1 and IgG2a subclass is common, indicating antigen-specific induction [27]. Although the antigen has not yet been identified the typical ANA pattern in sera from Hg-treated BALB/c mice is fine-speckled with a distinct staining of nuclear membrane and of condensed chromosomes in dividing cells [27].

Mesangial cells express FcγRs and may mediate binding of IC by recognition of exposed Fc-part [41]. The loss of functionally activating FcγRs will hinder this particular mechanism of deposition as shown in the present study as well as in experimental autoimmune myasthenia gravis. FcγRIII deficient mice show reduction of immune deposits in neuromuscular junctions [42]. An additional consequence of lost FcγRs function is retarded CIC clearance [31], [32] leading to further accumulation of CIC in the circulation as seen in the present study.

In glomerular diseases IgG-IC may cause tissue damage due to FcγR activation as well as complement activation [43]. In an experimental model of IC-mediated disease induced by intravenous injection of soluble IgG-IC, Stokol *et* al showed that the damaging effect of IC deposits was primarily dependent on C1q, followed by neutrophil recruitment [44]. The effect of renal mesangial and systemic vascular IC deposits in Hg-treated BALB/c mice is a mild glomerular endocapillary cell proliferation and slight widening of the mesangium, neither of which is affected by the loss of activating FcγRs, or by vasculitis [27]. How might this mild histological reaction be explained? The frequencies and/or the amounts of the strongly complement-activating IgG2b, IgG3, and especially IgG2a antibodies [45] are low in the IC deposits of HgIA. Instead the IgG1 isotype, which does not activate complement via the classical pathway, dominates [27] as shown also by the lack of C1q in the deposits (present study). Another explanation for the mild renal reaction in HgIA might be that the glomerular IC deposits in HgIA are strictly localised to the mesangium [46], which leads to less histological damage [47]. In fact, when the spontaneously developing glomerular IC-deposits of NZB/WF1 mice, preferentially situated in the capillary loops, are relocalised to the mesangium, the histological damage is greatly reduced [48]. IgG-Fc modulation by *in vivo* administration of endoglycosidase-S is a novel and fascinating possibility to treat autoantibody-/IC-mediated diseases [49]. In the future, other strategies to specifically interfere with FcγR-mediated IC handling may also become options to treat IC-mediated disease.

In conclusion, mice deficient regarding the function of activating FcγRs respond to an Hg-induced autoimmune stimulus with increased levels and altered quality of CIC compared with mice expressing the intact receptors. The lack of increased IC deposits in target organs for tissue IC deposits and the reduced uptake of CIC in the liver speak in favour of a reduced elimination of CIC but at the same time protection from a more pronounced histological kidney damage.

## Methods

### Animals and housing

Female BALB/c mice with a targeted knockout mutation for the signal-transducing (ITAM-containing) intracellular γ-chain dimer (FcRγ^−/−^), causing a functional loss of the activating FcγRs, were obtained from Taconic M&B (Georgetown, NY, USA). Corresponding BALB/c mice without a mutation (wild type - wt mice) were obtained from Taconic M&B (Ry, Denmark). All mice were 11–14 weeks old at onset of the experiments except for the animals in the studies of blood clearance and tissue uptake of CIC, where the mice were 39–44 weeks old. The mice were kept under specific pathogen-free conditions and housed under 12-hour dark-/12-hour light cycles in steel-wire cages. They were fed with pellets (Transbreed E, Special Diets services, Witham, UK) and tap water *ad libitum*. The animal ethics committee in Linköping approved the study protocol (ID 14-06).

### Treatment

Groups of wt and FcRγ^−/−^ mouse strains were exposed to 15 mg/L HgCl_2_ (Fluka Chemie, Buchs, Germany) in the sterilised drinking water given *ad libitum* for 26–35 days. Control mice received sterilised tap water only.

### Blood and tissue sampling

Blood samples were obtained from the retro-orbital vein plexus.

The first groups of wt and FcRγ^−/−^ mice were bled before onset of treatment and then after 12, 18, 26, and 35 days. At sacrifice after 35 days treatment samples of the kidney and spleen were obtained for examination of tissue IC deposits. The blood was allowed to clot at 4°C for 2 hours, and CIC measured using these fresh sera.

The second groups of wt and FcRγ^−/−^ mice were treated with Hg for 12–35 days in order to assess the specific time needed for formation of tissue IC deposit. Pieces of the kidney and spleen were collected at sacrifice after 12, 14, 16, 18, 26, and 35 days of treatment in wt mice, and after 12, 18, and 26 days of treatment in FcRγ^−/−^ mice.

The third group of wt and FcRγ^−/−^ mice were treated with Hg for 15–17 days and then given a single tail vein injection of 0.2 ml preformed isotope-labelled soluble ICs (see below) at concentration of 0.7 mg/ml to analyse blood clearance and liver uptake of CIC. Blood samples (50 µl) were taken after 0, 2, 4, 6, 8, 12, 22 and 32 minutes following the injection and the liver was collected at sacrifice.

### Assessment of circulating immune complexes

#### Circulating immune complexes assessed by PEG precipitation followed by analyses of IgG1, IgG2a and IgG2b ELISA

CIC in serum was measured by polyethylene glycol (PEG)-induced precipitation of immune complexes [12], [50]. Equal volumes of serum and 8% PEG (Fluka) were incubated at 4°C for 1 h and then centrifuged at 1000 g for 1 h. The pellet was washed, resuspended in phosphate-buffered saline (PBS pH 7.4) and stored at −20°C. The method used for detection of IgG1 has been described before [27]. A standard curve using mouse myeloma protein of the IgG1 (LO-IMEX) isotype was used to obtain the actual concentration. The IgG2a and IgG2b content were measured by a commercially available enzyme-linked immunosorbent (ELISA) kit (Bethyl Laboratories Inc, Montgomery, Texas, USA). To obtain the actual IgG2a or IgG2b concentration the standard supplied with the kit was used.

#### Circulating immune complexes assessed by a C1q-binding assay

CIC containing C1q were measured using a mouse specific ELISA kit from Alpha Diagnostic International (San Antonio, TX, USA). Briefly, serum was added to wells pre-coated with C1q. A horseradish-peroxidise- (HRP-) conjugated anti-mouse IgG detection antibody (ab) was added followed by substrate buffer. After adding stop solution the absorbance was measured at 450 nm and background values were subtracted. Positive and negative controls included in the kit gave the expected results.

### Assessment of tissue immune complex deposits

Pieces of the left kidney and the spleen were examined for IC deposits with direct immunofluorescence microscopy as described before [27]. Briefly, snap frozen tissue pieces were sectioned and incubated with either a fluorescein-isothiocyanate- (FITC-) conjugated goat anti-mouse ab against the IgG1, IgG2a, IgG2b or IgG3 isotype (Southern Biotechnology, Birmingham, AL, USA), C1q (Cedarlane, Burlington, Canada), or C3c (Organon-Technica, West Chester, PA, USA). Kidneys from aged NZB/WF1 mice were used as a positive control. The presence of deposits in glomeruli, renal and splenic vessel walls was examined with a fluorescence microscope (Nikon, Tokyo, Japan). The endpoint titre for the IgG isotypes, C1q and C3c was defined as the highest dilution of detection ab giving a specific staining of the tissue. No staining at an ab dilution of 1∶40 was considered as negative and given the value 0. The amount of the IgG isotypes, C1q and C3c in renal and splenic vessel walls was scored from 0–4 (0, no specific staining; 1, slight staining; 2, moderate staining; 3, strong staining and 4, very strong staining). All examinations were done without knowledge of treatment given or other results.

### Immune complex formation and isotope labeling

DNP-conjugated HSA was prepared essentially as described previously [51]. Briefly,1 g of HSA (Sigma, St Louis, Missouri, USA) and 1 g potassium carbonate was dissolved in distilled water and allowed to react with 1 g 2,4-dinitrobenzene sulfonate in the dark, at 37°C under continuous agitation, for approximately 5 h to achieve DNP-HSA with a conjugation degree of 4–6 DNP per HSA. The DNP-HSA protein was then passed through a Sephadex G-10 column and dialysed against distilled water at 4°C for 24 h, lyophilized and stored at 4°C.

### ^125^I-TC labelling and CIC formation

The radioactive tyramine cellbiose (TC) label has the advantage of remaining intracellularly over a long time after endocytosis and degradation, allowing analysis of accumulated intracellular uptake of labelled IC. Radiolabelling of HSA was accomplished by the TC method as previously described [52] with some modifications. In brief, iodinated TC (^125^I-TC) was prepared by reacting TC (6 µl of 10 mM solution in PBS) with Na^125^I (1.2 mCi, Perkin-Elmer) in Iodo-Gen tubes (Pierce) for 30 min at room temperature and was then activated by transferring the solution to a tube containing cyanuric chloride (6 µl of 1.8 mg solution in acetone) and potassium iodide (6 µl of 0.1 M solution) for 3 min. The activated ^125^I-TC adduct was then covalently coupled to DNP-HSA (0.5 mg in 200 µl 10 mM borate buffer, pH 8.8). To remove unincorporated ^125^I-TC, the labelled protein (^125^I-TC-DNP-HSA) was passed through a Sephadex G-10 column (GE Healthcare) eluated with PBS. Radiolabelled preparations were>95% trichloroaetic acid-precipitable. Specific activities obtained were in the range of 7–8×10^5^ cpm/µg. The ^125^I-TC moiety formed after degradation of ^125^I-TC-labeled proteins is not released to the medium but remains trapped in degradative compartments [52], thus enabling assessment of the accumulated uptake of radioactive protein over time. ^125^I-TC-labelled DNP-HSA was diluted with PBS and allowed to react with polyclonal rabbit IgG anti-DNP ab (AbD Serotec, Oxford, England) at 4-fold ab excess for 45 min at 37°C.

### Statistical methods

Differences between the groups were analysed by the non-parametric Mann-Whitney test or Fisher's exact test. *P<0.05* was considered statistically significant.
